# Molecular identification and subtyping of *Blastocystis* sp. in laboratory rats in China

**DOI:** 10.1051/parasite/2020035

**Published:** 2020-05-15

**Authors:** Junqiang Li, Yueyue Yuan, Yuxi Jiang, Wen Wang, Liqin Chao, Ruiqin Sun, Jun Li, Md Robiul Karim, Meng Qi

**Affiliations:** 1 Academy of Chinese Medical Sciences, Henan University of Chinese Medicine 450046 Zhengzhou PR China; 2 College of Animal Science, Tarim University Alar 843300 Xinjiang PR China; 3 Faculty of Veterinary Medicine and Animal Science, Bangabandhu Sheikh Mujibur Rahman Agricultural University 1706 Gazipur Bangladesh

**Keywords:** *Blastocystis* sp., Identification, Subtype, Laboratory rats

## Abstract

*Blastocystis* sp. is a ubiquitous protist that has been frequently reported in humans and animals worldwide. A total of 355 fecal samples of experimental rats were collected from four laboratory rearing facilities in China, and *Blastocystis* sp. was detected by PCR amplification of the partial small subunit ribosomal (SSU) rRNA gene. Twenty-nine (8.2%, 29/355) samples were positive for *Blastocystis* sp., with the highest infection rate (20.7%, 24/116) in rats of the Zhengzhou1, followed by that in the Zhengzhou2 (5.0%, 2/40), Shenyang (3.0%, 3/100) and Wuhan (0) rearing facilities. Among the three rat strains, Sprague–Dawley (SD) rats had higher infection rates (11.3%, 17/151) compared to Wistar rats (8.7%, 9/104) and spontaneously hypertensive (SH) rats (3.0%, 3/100). Two *Blastocystis* sp. subtypes (ST4 and ST7) were identified. ST4 was the predominant subtype detected in 26 samples (89.7%). A phylogenetic analysis demonstrated that the sequences of ST4 and ST7 obtained in this study were clustered with their reference subtypes. To our knowledge, this is the first report of *Blastocystis* sp. in experimental rats in China. Pathogen infections in laboratory animals need to be monitored due to fecal-oral transmission.

## Abstract (fr)

*Blastocystis* sp. est un protiste omniprésent qui a été fréquemment signalé chez l’homme et les animaux du monde entier. Un total de 355 échantillons fécaux de rats de laboratoire ont été prélevés dans quatre installations d’élevage en laboratoire en Chine et *Blastocystis* sp. a été détectée par amplification par PCR du gène partiel de la petite sous-unité de l’ARNr ribosomique (SSU). Vingt-neuf échantillons (8,2 %, 29/355) étaient positifs pour *Blastocystis* sp., avec le taux d’infection le plus élevé (20,7 %, 24/116) chez les rats des élevages de Zhengzhou1, suivi de ceux de Zhengzhou2 (5,0 %, 2/40), Shenyang (3,0 %, 3/100) et Wuhan (0). Parmi les trois souches de rats, les rats Sprague-Dawley (SD) avaient un taux d’infection plus élevé (11,3 %, 17/151) que les rats Wistar (8,7 %, 9/104) et les rats spontanément hypertendus (SH) (3,0 %, 3/100). Deux sous-types de *Blastocystis* sp. ont été identifiés, ST4 et ST7. ST4 était le sous-type prédominant, détecté dans 26 échantillons (89,7 %). Une analyse phylogénétique a démontré que les séquences de ST4 et ST7 obtenues dans cette étude étaient groupées avec leurs sous-types de référence. À notre connaissance, il s’agit du premier signalement de *Blastocystis* sp. chez des rats de laboratoire en Chine. Les infections pathogènes chez les animaux de laboratoire doivent être surveillées en raison de la transmission fécale-orale.

## Introduction

*Blastocystis* sp. is a prevalent protist that has been frequently reported in humans and animals worldwide [20]. Molecular phylogenetic analysis based on small subunit (SSU) rDNA sequences demonstrated that *Blastocystis* is placed within the Stramenopiles [11]. Genetic heterogeneity of the SSU rRNA gene reveals 17 recognized subtypes (ST1–ST17) of *Blastocystis* sp.; humans can host ST1–ST9 and 12, and more than 90% of human *Blastocystis* strains belong to ST1–ST4 [22].

Experimental rats are a variant of the brown rat (*Rattus norvegicus*) that has been used as an animal experimental model through artificial rearing in the laboratory since the 1850s [7]. Nowadays, experimental rats are widely used in biomedical research because of their strong fecundity, clear and consistent genetic characteristics, and similar experimental reaction conditions [8]. Infections with additional pathogens, such as unrelated parasites, can be a persistent constraint for animal breeding and experimentation due to developmental interference, which may impact the progress of biomedical research [10, 19]. *Blastocystis* sp. infect the intestinal lamina propria, resulting in mucus membrane sloughing and inflammatory cell infiltration in the laboratory rats [5], which might have a strong negative impact on the results of intestinal developmental biology experiments.

To date, except for a few data on *Blastocystis* sp. infections in wild rodents [6, 15], there is a knowledge gap concerning this infection and subtype identification in laboratory rats. The aim of this study was to determine the prevalence of *Blastocystis* sp. and the subtype distributions in laboratory rats in China.

## Materials and methods

### Ethics statement

Ethical clearance was obtained from the Institutional Committee on Animal Care and Use in Research (ICACUR) of Henan University of Chinese Medicine (License no.: DWLL201906003). Appropriate permission was obtained and the collection protocol was reviewed and approved by the head of each rearing facility before fecal sample collection from the laboratory rats.

### Animal housing and sample collection

The study was conducted in four experimental rat rearing facilities in China (two in Zhengzhou City, one in Shenyang City, and one in Wuhan City) in July and August 2019. All the four facilities had been rearing laboratory rats for more than five years, with an overall production of 1000–3000 rats per year (2019 data). The rats were reared in a strict management regime in the rearing facilities and had no opportunity for contact with the wild rats. The experimental rats were first introduced to the rearing facilities from other universities (facilities) and/or from animal dealers with laboratory animal quality certification.

The experimental rats were housed in a cage with 1–3 rats, and all deposits from each cage were pooled as one sample, as previously described [9]. A total of 355 fresh fecal samples were collected from the experimental rats, including 104 from Wistar rats, 151 from Sprague-Dawley (SD) rats, and 100 from spontaneously hypertensive (SH) rats. All the studied rats were adult females that showed no obvious signs of diarrhea during sampling. Approximately 3 g of each fecal sample were collected in a sterile plastic zippered bag, marked with relevant information, and shipped to the laboratory under cool conditions. To avoid contamination, each sample was collected with separate sterile disposable hand gloves. The fecal samples were stored at 4 °C, and DNA was extracted within 72 h.

### DNA extraction

Total genomic DNA from each fecal sample (approximate 200 mg) was extracted with an E.Z.N.A.^®^ Stool DNA Kit (Omega Bio-tek Inc., Norcross, Georgia, USA), according to the manufacturer’s recommended protocol. The extracted DNA was stored at −20 °C until PCR amplification.

### PCR amplification and sequence analysis

*Blastocystis* sp. was detected by using the following primers: forward F505–532 (5′ – GGA GGT AGT GAC AAT AAA TC – 3′) and reverse R998–1017 (5′ – TGC TTT CGC ACT TGT TCA TC – 3′) that amplified a 479 bp variable region of the SSU rRNA gene of the parasite [18]. 2× EasyTaq PCR SuperMix (TransGen Biotech Co., Beijing, China) was used for the PCR amplification, with DNA from human-derived genotype ST1 as the positive control and distilled water without any DNA as the negative control. The positive PCR amplicons were transfer to a commercial sequencing company (GENEWIZ, Suzhou, China), and the sequence accuracy was confirmed with bidirectional sequencing.

### *Blastocystis* sp. sequences and phylogenetic analysis

The obtained sequences were aligned by BLAST (Basic Local Alignment Search Tool) (https://blast.ncbi.nlm.nih.gov/) to determine the *Blastocystis* sp. positive sequences. The *Blastocystis* sp. subtypes were identified by an online platform: *Blastocystis* locus/sequence definitions database (https://pubmlst.org/bigsdb?db=pubmlst_blastocystis_seqdef). The subtypes of *Blastocystis* sp. obtained from this study were compared with the known subtypes with maximum likelihood (ML) and neighbor-joining (NJ) analyses with aligning by Muscle in the Mega 7 program (http://www.megasoftware.net/). A bootstrap method was used to assess the robustness of the clusters using 1000 replicates.

The representative *Blastocystis* sp. nucleotide sequences identified in rats were submitted to GenBank at the National Center for Biotechnology Information under accession numbers: MT071884–MT071888.

## Results and discussion

*Blastocystis* sp. was first identified in laboratory rats (*Rattus norvegicus*) in Singapore in 1997 [1]. In the present study, PCR examination of 355 fecal samples collected from experimental rats indicated that 8.2% of samples (29/355) were positive for *Blastocystis* sp., which is higher than that in house rats (6.1%, 6/98) in East Java (Indonesia) [13], as well as in captive brown rats (3.7%, 4/108) in northeastern China [24]. Rats from three rearing facilities out of four were positive for *Blastocystis* sp., with Zhengzhou1 having the highest infection rate (20.7%, 24/116), followed by Zhengzhou2 (5.0%, 2/40) and Shenyang (3.0%, 3/100), and no positive sample was detected in the Wuhan facility. Among the three rat strains, the highest infection rate of 11.3% (17/151) was found in SD rats, followed by Wistar rats (8.7%, 9/104) and SH rats (3.0%, 3/100) (Table 1). A previous study reported that *Blastocystis* cysts are transmitted by the fecal-oral route [25]. Water or food contamination with the cysts might lead to higher infection rates in intensive rearing conditions. Thus, the high occurrence of *Blastocystis* sp. in the experimental rats in this study implies poor hygiene management of these animals in the three rearing facilities. However, the absence of *Blastocystis* infection in the Wuhan facility might be explained by the implementation of strict hygiene practices in the management of rats that could possibly curtail the transmission of the parasitic cysts.

**Table 1:** Occurrence and genotypic distributions of *Blastocystis* sp. in laboratory rats in China.

Parameters	Number of samples tested	Positive samples (%)	*Blastocystis* sp. subtype (no. of isolates)
Rearing facilities			
Zhengzhou1	116	24 (20.7%)	ST4 (23); ST7 (1)
Zhengzhou2	40	2 (5.0%)	ST4 (2)
Shenyang	100	3 (3.0%)	ST4 (1); ST7 (2)
Wuhan	99	0	
Rat breeds			
Wistar rats	104	9 (8.7%)	ST4 (9)
SD rats	151	17 (11.3%)	ST4 (16); ST7 (1)
SH rats	100	3 (3.0%)	ST4 (1); ST7 (2)
Total	355	29 (8.2%)	ST4 (26); ST7 (3)

*Note*: SD: Sprague Dawley; SH: Spontaneously hypertensive.

Two *Blastocystis* sp. subtypes (ST4 and ST7) were identified in the present study, with ST4 being the predominant subtype (89.7%, 26/29). Two kinds of ST4 sequences (ST4a and ST4b) were identified, with three nucleotide substitutions in the ST4b sequence at the 189 (A–G), 211 (G–A), and 352 (A–T) positions compared to the ST4a sequence. The ST4a sequence (*n* = 25) showed 100% homology with an isolate from a human sample in the United States (JN682513), and isolates from rats in Indonesia (MH127480), Japan (MH127497), and Norway (AB071000). The ST4b sequence (*n* = 1) was identical to the isolates obtained from humans in Japan (AY244621) and Germany (AY244620), and an isolate from Wistar rats in France (AY590114). On the other hand, the ST7 sequences were of three types, such as ST7a, ST7b, and ST7c. The ST7a sequence was 94.7% homologous to an isolate from chickens in India (MG720557). Both the ST7b and ST4c sequences were 98.9% similar to an isolate from the swan goose (*Anser cygnoides*) in Japan (AB107973).

Previous data have shown that *Blastocystis* sp. subtypes ST1, ST2, ST3, ST4, and ST7 infect rats experimentally [4, 17]. However, a molecular survey identified subtype ST4 as the only subtype in wild rodents (*Rattus novercious* and *R. exulans*) in Indonesia (*n* = 12) and Japan (*n* = 11) [6], and the Brown rat (*Mus musculus*) in Heilongjiang (*n* = 4), China [24]. In another study, subtype ST2 (*n* = 3) was the only subtype in rodents (*Rattus rattus*) in Colombia [15]. To sum up, among the subtypes of *Blastocystis* sp. infecting rats, subtype ST4 was the most prevalent. Besides in rodents, subtype ST4 is one of the most common subtypes found in humans [14]. Similarly, subtype ST4 has also been reported in non-human primates, giraffes, kangaroos, dogs, snow leopards, and ostriches [2, 15, 16, 21, 23]. A study by Iguchi et al. [5] demonstrated pathogenicity of subtype ST4 in rats experimentally colonized by *Blastocystis* from reservoir rats. However, ST4 is relatively common in the gut of healthy individuals in Europe, suggesting the subtype as a component of healthy gut microbiome [15]. It is reported that subtype ST7 commonly infects rats [4], birds, [24], and humans [3]. A few previous studies have attempted to infer the pathogenicity of subtype ST7 in some hosts. In such a study, subtype ST7 had been shown to decrease the levels of diversity and richness of beneficial gut bacteria, such as Bifidobacterium and *Lactobacillus*, of the host individuals [22]. In another study, proteases released in culture supernatants of subtype ST7 were shown to be able to cleave human-secreted immunoglobulin A, and thus affected the immune response of the host [12]. However, in this study, no clinical symptoms were observed in the rats infected with subtype ST7.

**Figure 1:**
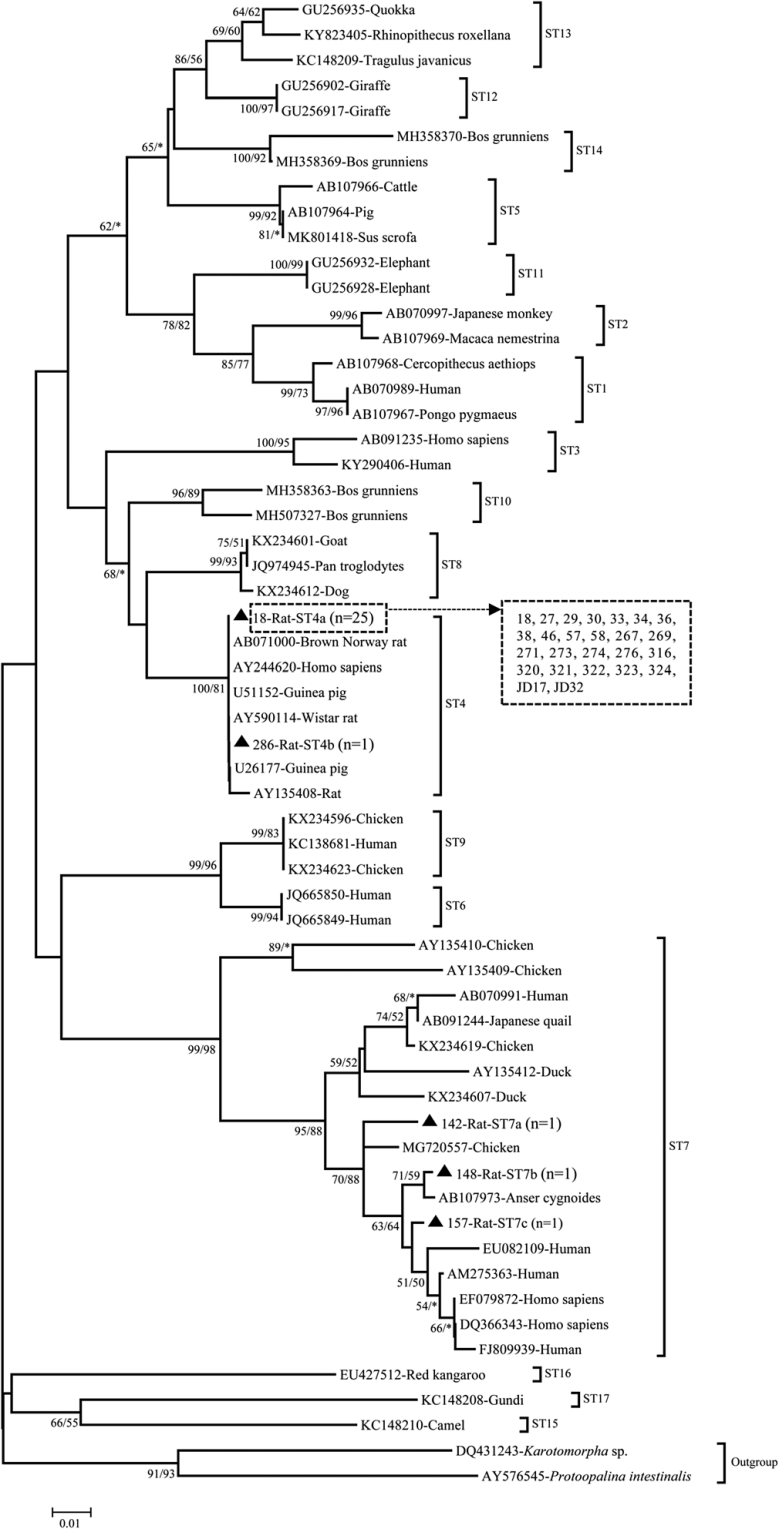
Phylogenetic relationships of the *Blastocystis* sp. subtypes identified in the present study and other reference subtypes. The phylogeny was inferred with both neighbor-joining (NJ) and maximum likelihood (ML) analysis. Evolutionary distances were computed using the maximum composite likelihood method with rate variation among sites modeled using a gamma distribution (shape parameter = 0.5). The percentage of trees that clustered together based on a bootstrap test (1000 replicates) and posterior probabilities (expressed as a percentage) are shown beside the branch nodes. An asterisk indicates a value of less than 50%; if both analyses produced values lower than 50%, no values are shown for that node. The genotypes detected in present study in bold are shown with filled triangles.

Phylogenetic analysis using five representative sequences and 54 reference sequences of *Blastocystis* sp. demonstrated that the sequences of subtypes ST4 and ST7 obtained in the present study clustered with their reference subtypes (Fig. 1). Based on the constructed tree, the sequences of ST4a and ST4b clustered with each other with high bootstrap support. Meanwhile, the sequences of ST7a, ST7b, and ST7c clustered within the reference sequences of ST7.

## Conclusions

To our knowledge, this is the first report of *Blastocystis* sp. in laboratory rats in China. The results of this study showed that 8.2% of the rats were infected with either subtype ST4 or ST7 of *Blastocystis* sp., which might interfere with biological experiments in these rats. Therefore, parasitic infection in laboratory rats should be monitored on a regular basis.

## References

[1] Chen XQ, Singh M, Ho LC, Tan SW, Ng GC, Moe KT, Yap EH. 1997 Description of a *Blastocystis* species from *Rattus norvegicus*. Parasitology Research, 83(4), 313–318. 9134551 10.1007/s004360050255

[2] Cian A, El Safadi D, Osman M, Moriniere R, Gantois N, Benamrouz-Vanneste S, Delgado-Viscogliosi P, Guyot K, Li L, Monchy S, Noël C, Poirier P, Nourrisson C, Wawrzyniak I, Delbac F, Bosc S, Chabé M, Petit T, Certad G, Viscogliosi E. 2017 Molecular epidemiology of *Blastocystis* sp. in various animal groups from two French zoos and evaluation of potentially zoonotic risk. PLoS One, 12(1), e0169659. 28060901 10.1371/journal.pone.0169659 PMC5217969

[3] Forsell J, Granlund M, Stensvold CR, Clark CG, Evengard B. 2012 Subtype analysis of *Blastocystis* isolates in Swedish patients. European Journal of Clinical Microbiology and Infectious Diseases, 31(7), 1689–1696. 22350386 10.1007/s10096-011-1416-6

[4] Iguchi A, Ebisu A, Nagata S, Saitou Y, Yoshikawa H, Iwatani S, Kimata I. 2007 Infectivity of different genotypes of human *Blastocystis hominis* isolates in chickens and rats. Parasitology International, 56, 107–112. 17251054 10.1016/j.parint.2006.12.004

[5] Iguchi A, Yoshikawa H, Yamada M, Kimata I, Arizono N. 2009 Expression of interferon gamma and proinflammatory cytokines in the cecal mucosa of rats experimentally infected with *Blastocystis* sp. strain RN94-9. Parasitology Research, 105(1), 135–140. 19255785 10.1007/s00436-009-1373-5

[6] Katsumata M, Yoshikawa H, Tokoro M, Mizuno T, Nagamoto T, Hendarto J, Asih PBS, Rozi IE, Kimata I, Takami K, Syafruddin D. 2018 Molecular phylogeny of *Blastocystis* isolates from wild rodents captured in Indonesia and Japan. Parasitology Research, 117(9), 2841–2846. 29968038 10.1007/s00436-018-5973-9

[7] Krinke GJ. 2000 History, strains, and models, in The Laboratory Rat (Handbook of Experimental Animals), Bullock G, Bunton T, Editors. Academic Press: Waltham, MA.

[8] Lazar J, Moreno C, Jacob HJ, Kwitek AE. 2005 Impact of genomics on research in the rat. Genome Research, 15(12), 1717–1728. 16339370 10.1101/gr.3744005

[9] Li J, Jiang Y, Wang W, Chao L, Jia Y, Yuan Y, Wang J, Qiu J, Qi M. 2020 Molecular identification and genotyping of *Enterocytozoon bieneusi* in experimental rats in China. Experimental Parasitology, 3, 107850. 10.1016/j.exppara.2020.107850 32027893

[10] Lytvynets A, Langrova I, Lachout J, Vadlejch J. 2013 Detection of pinworm eggs in the dust of laboratory animals breeding facility, in the cages and on the hands of the technicians. Laboratory Animals, 47, 71–73. 23230226 10.1258/la.2012.012060

[11] Noël C, Peyronnet C, Gerbod D, Edgcomb VP, Delgado-Viscogliosi P, Sogin ML, Capron M, Viscogliosi E, Zenner L. 2003 Phylogenetic analysis of *Blastocystis* isolates from different hosts based on the comparison of small-subunit rRNA gene sequences. Molecular and Biochemical Parasitology, 126(1), 119–123. 12554093 10.1016/s0166-6851(02)00246-3

[12] Poirier P, Wawrzyniak I, Vivarès CP, Delbac F, El Alaoui H. 2012 New insights into *Blastocystis* spp.: a potential link with irritable bowel syndrome. PLoS Pathogens, 8(3), e1002545. 22438803 10.1371/journal.ppat.1002545 PMC3305450

[13] Prasetyo RH. 2016 Survey of house rat intestinal parasites from Surabaya District, East Java, Indonesia that can cause opportunistic infections in humans. Southeast Asian Journal of Tropical Medicine and Public Health, 47(2), 194–198. 27244955

[14] Qi M, Wei Z, Zhang Y, Zhang Q, Li J, Zhang L, Wang R. 2020 Genetic diversity of *Blastocystis* in kindergarten children in southern Xinjiang, China. Parasites & Vectors, 13(1), 15. 31924261 10.1186/s13071-020-3890-0 PMC6954523

[15] Ramírez JD, Sánchez LV, Bautista DC, Corredor AF, Flórez AC, Stensvold CR. 2014 *Blastocystis* subtypes detected in humans and animals from Colombia. Infection Genetics and Evolution, 22, 223–228. 10.1016/j.meegid.2013.07.020 23886615

[16] Roberts T, Stark D, Harkness J, Ellis J. 2013 Subtype distribution of *Blastocystis* isolates from a variety of animals from New South Wales, Australia. Veterinary Parasitology, 196, 85–89. 23398989 10.1016/j.vetpar.2013.01.011

[17] Růžková J, Květoňová D, Jirků M, Lhotská Z, Stensvold CR, Parfrey LW, Jirků Pomajbíková K. 2018 Evaluating rodent experimental models for studies of *Blastocystis* ST. Experimental Parasitology, 191, 55–61. 29959915 10.1016/j.exppara.2018.06.009

[18] Santín M, Gómez-Muñoz MT, Solano-Aguilar G, Fayer R. 2011 Development of a new PCR protocol to detect and subtype *Blastocystis* spp. from humans and animals. Parasitology Research, 109(1), 205–212. 21210149 10.1007/s00436-010-2244-9

[19] Sousa JE, Carvalho EF, Levenhagen MA, Chaves LA, Costa-Cruz JM. 2016 Diagnosis of the pinworm *Syphacia muris* in the Wistar rat *Rattus norvegicus*. Journal of Helminthology, 90(1), 117–120. 25327496 10.1017/S0022149X14000753

[20] Stensvold CR, Clark CG. 2016 Current status of *Blastocystis*: a personal view. Parasitology International, 65, 763–771. 27247124 10.1016/j.parint.2016.05.015

[21] Stensvold CR, Alfellani MA, Nørskov-Lauritsen S, Prip K, Victory EL, Maddox C, Nielsen HV, Clark CG. 2009 Subtype distribution of *Blastocystis* isolates from synanthropic and zoo animals and identification of a new subtype. International Journal for Parasitology, 39, 473–479. 18755193 10.1016/j.ijpara.2008.07.006

[22] Stensvold CR, Tan KSW, Clark CG. 2020 Blastocystis. Trends in Parasitology, 36(3), 315–316. 32001134 10.1016/j.pt.2019.12.008

[23] Wang W, Cuttell L, Bielefeldt-Ohmann H, Inpankaew T, Owen H, Traub RJ. 2013 Diversity of *Blastocystis* subtypes in dogs in different geographical settings. Parasites & Vectors, 6, 215. 23883734 10.1186/1756-3305-6-215 PMC3734043

[24] Wang J, Gong B, Liu X, Zhao W, Bu T, Zhang W, Liu A, Yang F. 2018 Distribution and genetic diversity of *Blastocystis* subtypes in various mammal and bird species in northeastern China. Parasites & Vectors, 11(1), 522. 30236147 10.1186/s13071-018-3106-z PMC6148767

[25] Yoshikawa H, Yoshida K, Nakajima A, Yamanari K, Iwatani S, Kimata I. 2004 Fecal-oral transmission of the cyst form of *Blastocystis hominis* in rats. Parasitology Research, 94(6), 391–396. 15480786 10.1007/s00436-004-1230-5

